# Shifting the Standard of Beauty: Beginning of the Body Inclusive Model

**DOI:** 10.7759/cureus.25584

**Published:** 2022-06-01

**Authors:** Jaclyn B Anderson, Melissa R Laughter, Jonny Hatch, Payal Patel, Mayra Maymone, Neelam A Vashi

**Affiliations:** 1 Department of Dermatology, University of Colorado School of Medicine, Denver, USA; 2 Medicine, Texas College of Osteopathic Medicine, University of North Texas Health Science Center, Fort Worth, USA; 3 Department of Dermatology, University of Illinois at Chicago, Chicago, USA; 4 Department of Dermatology, Brown University, Providence, USA; 5 Department of Dermatology, Boston University School of Medicine, Boston, USA

**Keywords:** plus-size models, perception of beauty, body image, waist-hip ratio, social media

## Abstract

Background: While the American standard of beauty idolizes unattainable thinness, social media exposure has been instrumental in crafting a more inclusive perception of beauty.

Methods: Using several websites with public data on models, we gathered body measurements and characteristics of both plus-size and the overall top 10 paid mainstream models. We then collected social media data for these models using the social media analytics tool called Social Blade. We compared social media data between plus-size and mainstream models.

Results: While plus-size models have increased BMI, the waist/hip ratio was 0.74 on average, compared to 0.71 in mainstream models. The average social media following among the top 10 plus-size models was 3.8 million compared to 38 million amongst the top 10 mainstream models (p = 0.039). There was no significant difference between the average likes per post, average comments per post, and total posts between the top mainstream models and top plus-size models (p-values 0.11, 0.12, and 0.15, respectively).

Conclusion: With the changing societal body image in America, plus-size models have gained in popularity and positively impacted a body-inclusive model of beauty. However, the mainstream model still prevails as the social media powerhouse of influence.

## Introduction

The perception of the ideal body size has historically shifted over the years and varied across cultures. The American standard of beauty appears to idolize unattainable thinness as seen in Victoria's Secret models and their diminishing body size [1]. Social media exposure has become instrumental in the evolution and creation of beauty standards dichotomously leading to social dysmorphia [2] while also being instrumental in crafting a more inclusive perception of beauty. In this study, we explored the impact of plus-size models by reviewing their body size metrics and social media presence to better understand their emerging role in the current standards of beauty.

## Materials and methods

A list of the top plus-size models was compiled using a composite of several websites [3,4]. Using The Fashion Model Directory (FMD), a professional source of fashion information, and various modeling agency websites, the following information was extracted for all plus-size models: eye color, hair color, height, bust measurement, waist measurement, dress size, and shoe size. Data pertaining to social media presence (number of followers, average number of likes, average number of comments, and number of posts) was collected for each model using Social Blade, a social media database and analytics platform [5]. For comparison, the top 10 highest paid mainstream models were determined using Forbes, a well-established global media company that provides information on business and technology. Social media involvement of these models was collected in a similar fashion to plus-size models. A t-test was used to analyze the data. P-values less than 0.05 were considered significant. Institutional review board approval was waived by The University of Colorado Denver Institutional Review Board.

## Results

A total of 159 plus-size models and 10 mainstream models were included in this study (information for 23 of the plus-size models was not found). Overall group characteristics are shown in Table 1. Plus-size model height averaged 69 inches (in) (range: 60 inches to 73 inches, standard deviation (SD): 1.69), bust averaged 40 in the range: 30 inches to 52 inches, SD: 4.16, waist averaged 43 in the range: 24 inches to 49 inches, SD: 4.37, hips averaged 46 in the range: 32 inches to 60 inches, SD: 4.94, dress size averaged a size 14 (range: 0 to 26, SD: 4.01), and waist to hip ratio averaged 0.74 (range: 0.57 to 1, SD: 0.06). Hair colors included black (12%), brown (60%), blonde (23%), red (4%), and multi-colored (3%). Eye colors included brown (54%), blue (21%), green (14%), and hazel (12%).

**Table 1:** Characteristics of plus-size models

	n Value	Minimum	Maximum	Average	Standard Deviation
Height (in)	159	60	73	69	1.69
Bust (in)	154	32	52	40	4.05
Waist (in)	158	24	49	34	4.32
Hips (in)	156	32	60	46	4.89
Dress (US)	115	3	26	14	4.01
Waist/hip ratio	156	0.57	1	0.74	0.06

Social media involvement for all plus-size models and top 10 paid mainstream models is shown in Table 2 (social media accounts for 22 of the 159 models were not found). Among the plus-size models, AG had the largest number of followers with over 12 million. Collectively, 144 plus-size models had over 51 million followers and over 190,000 total posts. The top 10 most followed plus-size models were compared to the top 10 highest paid mainstream models in terms of social media engagement and following. The top 10 highest paid mainstream models averaged over 38 million followers compared to the top 10 plus-size models with an average of approximately 3.8 million followers (p = 0.039). There was no significant difference between the average likes per post, average comments per post, and total posts between the top mainstream models and top plus-size models (p-values 0.11, 0.12, and 0.15, respectively). 

**Table 2:** Instagram trends of top models and plus-size models Top models were determined based on a Forbes net worth review indicating these presented are 10 highest paid. The top 10 plus-size models were determined through review of all models included in our study and were included based on the highest number of followers. Metrics were determined using a social media analytics website called Social Blade [5]. An unpaired, two-tailed independent samples t-test was performed to determine statistical significance with a p-value of <0.05.

Top Model	Instagram Handle	Total Followers	Average Likes per Post	Average Comments per Post	Total Posts
Kendall Jenner (KJ)	@kendalljenner	154,473,887	5,676,560.00	23,702.20	3,027
Gigi Hadid (GH)	@gigihadid	64,410,746	5,303,170.00	21,560.40	3,141
Cara Delevingne (CD)	@caradelevingne	43,563,992	603,364.00	2,205.55	4,233
Bella Hadid (BH)	@bellahadid	39,178,750	897,137.00	2,167.90	2,738
Chrissy Teigen (CT)	@chrissyteigen	34,227,858	331,583.00	1,596.25	4,452
Gisele Bundchen (GB)	@gisele	16,995,512	663,354.00	3,709.05	693
Rosie Huntington-Whitely (RH)	@rosiehw	12,248,162	158,377.00	496.80	3,602
Karlie Kloss (KK)	@karliekloss	9,011,910	76,387.40	639.95	3,446
Doutzen Kroes (DK)	@doutzen	6,550,394	72,597.70	742.05	3,962
Joan Smalls (JS)	@joansmalls	3,716,862	20,638.60	184.45	2,650
Mean		38,437,807	1,380,317	5,700	3,194
Standard Deviation		45,236,932	2,187,076	8,999	1,068
Plus-Size Model					
Ashley Graham (AG)	@ashleygraham	12,126,147	297,653	1,675	3,352
Jordyn Woods (JW)	@jordynwoods	11,751,418	365,417	1,342	2,836
Iskra Lawrence (IL)	@iskra	4,600,297	112,875	399	1,724
Ashley Alexis Smith (AS)	@ashalexiss	2,226,086	28,984	317	284
Tess Holliday (TH)	@tessholliday	2,166,269	36,139	569	5,917
La’Tecia Thomas (LT)	@lateciat	1,536,621	57,146	612	198
Tabria Majors (TM)	@tabriamajors	1,501,581	84,545	961	769
Denise Bidot (DB)	@denisebidot	791,337	20,980	291	3,447
Hunter McGrady (HM)	@huntermcgrady	722,253	20,088	322	1,615
Barbie Ferreira (BF)	@barbieferreira	473,263	473,263	1,949	2,076
Mean		3,789,527	149,709	844	2,222
Standard Deviation		4,451,377	166,039	611	1,745
P-value		0.039	0.11	0.12	0.15

## Discussion

Over time, the presence of plus-size models has increased within the fashion industry. Societal pressures on retailers to incorporate an increased body diversity that fully represents their consumer base of society with an average dress size of 14 to 16 and BMI of 26.5 is likely contributory to the increasing presence of plus-size models [6,7]. This shift can be seen within the results of this study, as many of the top plus-size models had a significant social media presence. The average number of followers for top plus-size models was over 3.8 million. However, this pales in comparison to the social media presence and popularity of mainstream models. The top paid model within this category was KJ with an Instagram following of nearly half the population of the United States, with over 154 million followers. Importantly, the top 10 highest paid Forbes models do not include any plus-size models and can all be considered “runway” sized between dress sizes 0-4. Of note, models in both categories may gain additional celebrity from tv shows, movies, etc., which further contribute to their followings.

These results are consistent with those of previous studies showing that popular mainstream models (with an average Bust-Waist-Hip of 32.9-23.9-34.5) do not represent the body of the average American woman (with an average bust-waist-hip of 38-32-41) [8]. Furthermore, popular Victoria’s Secret models have in fact decreased in bust, waist, hip, and dress size over the past 23 years, contradictory to the trend seen amongst the US population [1]. Certain studies have shown that plus-size models are viewed more critically and subjected to increased weight bias compared to their mainstream counterparts, particularly among males [9]. We uncovered plus-size models have a higher average waist-hip ratio (WHR) of 0.74 compared to mainstream models (0.69) and the universal body attractiveness WHR (0.7) [1,10]. WHR among the average women is 0.75-0.8 and increases with age [11]. While the fashion industry seems to continue the veneration of a thin unattainable body image ideal, there has also been an increase in body diverse campaigns to promote body acceptance and inclusion. Multiple studies have demonstrated that women who viewed these campaigns found them to be more uplifting and empowering while also positively impacting their self-esteem and mood [12,13]. However, plus-size and body-inclusive models require more societal acceptance to match the social media influence of thinner, mainstream top models. Prior to social media, mainstream media was primarily responsible for propagating certain trends; today, social media carries a large responsibility in the assemblage of certain beauty standards. Social media is created and received by society indicating that these beauty ideals are largely the result of our own influence and design. As such, the influence of thinner, mainstream top models prevails and perhaps will only fade with increased visual exposure to plus-size models. 

Limitations of our study include use of unreliable sources to obtain characteristics of plus-size models and missing information due to unavailability.

## Conclusions

With the changing societal body image in America, plus-size models have gained in popularity and positively impacted a body inclusive model of beauty. However, the mainstream model still prevails as the social media powerhouse of influence. Currently, tension exists between groups desiring greater body inclusivity on social media, and the actual social media popularity of plus-size models. As plus-size models grow in popularity, this tension is likely to decrease. Yet, even as the desire for plus-size models grows, the WHR of models remains the same. Further research needs to be done regarding the public acceptance of body types without an ideal WHR in the body inclusive model.
